# Recombinant *Salmonella* Expressing *Burkholderia mallei* LPS O Antigen Provides Protection in a Murine Model of Melioidosis and Glanders

**DOI:** 10.1371/journal.pone.0132032

**Published:** 2015-07-06

**Authors:** Dina A. Moustafa, Jennifer M. Scarff, Preston P. Garcia, Sara K. B. Cassidy, Antonio DiGiandomenico, David M. Waag, Thomas J. Inzana, Joanna B. Goldberg

**Affiliations:** 1 Department of Microbiology, Immunology and Cancer Biology, University of Virginia, Charlottesville, Virginia, United States of America; 2 Department of Infectious Diseases, MedImmune, LLC, Gaithersburg, Maryland, United States of America; 3 Bacteriology Division, United States Army Medical Research Institute of Infectious Diseases (USAMRIID), Fort Detrick, Maryland, United States of America; 4 Virginia-Maryland Regional College of Veterinary Medicine and Virginia Tech-Carilion School of Medicine, Virginia Tech, Blacksburg, Virginia, United States of America; 5 Department of Pediatrics, Emory University School of Medicine and Children’s Hospital of Atlanta, Inc., Atlanta, Georgia, United States of America; East Carolina University School of Medicine, UNITED STATES

## Abstract

*Burkholderia pseudomallei* and *Burkholderia mallei* are the etiologic agents of melioidosis and glanders, respectively. These bacteria are highly infectious via the respiratory route and can cause severe and often fatal diseases in humans and animals. Both species are considered potential agents of biological warfare; they are classified as category B priority pathogens. Currently there are no human or veterinary vaccines available against these pathogens. Consequently efforts are directed towards the development of an efficacious and safe vaccine. Lipopolysaccharide (LPS) is an immunodominant antigen and potent stimulator of host immune responses. *B*. *mallei* express LPS that is structurally similar to that expressed by *B*. *pseudomallei*, suggesting the possibility of constructing a single protective vaccine against melioidosis and glanders. Previous studies of others have shown that antibodies against *B*. *mallei* or *B*. *pseudomallei* LPS partially protect mice against subsequent lethal virulent *Burkholderia* challenge. In this study, we evaluated the protective efficacy of recombinant *Salmonella enterica* serovar Typhimurium SL3261 expressing *B*. *mallei* O antigen against lethal intranasal infection with *Burkholderia thailandensis*, a surrogate for biothreat *Burkholderia spp*. in a murine model that mimics melioidosis and glanders. All vaccine-immunized mice developed a specific antibody response to *B*. *mallei* and *B*. *pseudomallei* O antigen and to *B*. *thailandensis* and were significantly protected against challenge with a lethal dose of *B*. *thailandensis*. These results suggest that live-attenuated SL3261 expressing *B*. *mallei* O antigen is a promising platform for developing a safe and effective vaccine.

## Introduction

Melioidosis and glanders are two debilitating and often fatal diseases in humans and animals caused by *Burkholderia pseudomallei* and *B*. *mallei*, respectively. *B*. *pseudomallei* is endemic in the soils of South East Asia and Northern Australia and its occurrence has been reported in other tropical and subtropical regions [1]. Humans and animals can be infected by *B*. *pseudomallei* by direct inoculation from soil or water into skin abrasions or by inhalation [2,3]. Symptoms of melioidosis may be exhibited many years after exposure; and presentation is commonly associated with a change in immune status of the host [4]. Melioidosis may manifest as a chronic low grade infection or acute fulminant pneumonia, which can progress to fatal sepsis within 48 hours of first clinical onset [5]. Despite antibiotic therapy and the presence of high antibody titers in infected patients, the organism is capable of undergoing a latency phase and can reactivate years after the initial infection [6].

Glanders is a zoonotic disease caused by *B*. *mallei*, found in equines and solipeds. *B*. *mallei* occasionally infects humans, such as laboratory workers and those in close contact with infected animals [7]. Infection may result from contamination of wounds, abrasions, or breaks in mucous membranes, or inhalation of aerosols containing the bacterium [8]. The symptoms are dependent on the route of exposure and can range from localized cutaneous lesions to more generalized symptoms including fever, malaise, pneumonia, and sepsis. When properly diagnosed and treated, the fatality rate is 50%, but cases of untreated septicemia can result in fatality rates as high as 95% [1]. Glanders was effectively eradicated in North America and Western Europe in the 1950s by mass culling of infected animals. There have been no natural cases of glanders reported in the USA in over 60 years due to strict screening of all horses entering the United States. However it remains endemic in the equine populations of Africa, Asia, and Central and South America [9].

*B*. *pseudomallei* and *B*. *mallei* pose a significant threat to human and animal health, and there is legitimate concern that these bacteria could be misused as bioterrorism agents. In fact, *B*. *mallei* was weaponized and used in the US Civil War, World War I and II [10], it has been suggested that the former Soviet military used this agent in Afghanistan during the 1980s [11] and that they were also weaponizing *B*. *pseudomallei* [9]. Based on the historical use of these pathogens as agents of bioterror, and their prevalence in South East Asia and Northern Australia, there exists a legitimate need for a vaccine to protect at-risk populations from natural and acquired infections [12].

Both *B*. *pseudomallei* and *B*. *mallei* possess several virulence determinants including capsular polysaccharides (CPS), type III & VI protein secretion systems, and quorum sensing that play an important role in their intracellular lifestyle, evading the host immune response, and persistence *in vivo* [13]. In addition, several studies have demonstrated the structural and antigenic similarities of the lipopolysaccharides (LPS) of *B*. *pseudomallei* and *B*. *mallei* [14–16]. The high genetic and biochemical similarities between these two species suggest similar mechanisms underlying their virulence.

*Burkholderia thailandensis* is an environmental saprophyte that is closely related to *B*. *mallei* and *B*. *pseudomallei*, encompassing about 99% gene similarity. When first isolated, it was incorrectly identified as *B*. *pseudomallei* due to the similar characteristics of the two species [17]. A notable difference is the ability of *B*. *thailandensis* to assimilate L-arabinose, in contrast to *B*. *pseudomallei*, which lacks the entire arabinose-assimilation operon [18]. The LPS O antigen of *B*. *thailandensis* is structurally similar to these pathogenic species as well [16,19]. Considered avirulent and non-pathogenic to humans, *B*. *thailandensis* does not require strict biocontainment in comparison to other virulent *Burkholderia spp*. Thus, it is considered an attractive surrogate for studying various diagnostic aspects and as a model for vaccine development studies against *B*. *pseudomallei* and *B*. *mallei* [16,20–23].

Bacterial polysaccharides are known to be immunodominant and protective antigens to several infectious agents. A number of polysaccharide conjugate vaccines are currently licensed to combat serious infections, which include vaccines to *Neisseria meningitidis* (Menomune), *Streptococcus pneumonia* (PCV7), and *Haemophilus influenzae* type b (Hib) [24]. The ability to enhance the immunogenicity of polysaccharide antigens was introduced by conjugation of the polysaccharide to a protein carrier that generates T cell help. The resulting antibody response generated to the polysaccharide conjugate is long lived due to immunological memory [25]. Antibody responses to glycoconjugate vaccines are dominated by the IgG1 and IgG3 in mice, and affinity maturation can be demonstrated over time [26].

Surface polysaccharides of *B*. *pseudomallei* have been investigated as subunit vaccines [27], and passive immunization with antibodies to LPS or CPS have been demonstrated to reduce the lethality of infection and increase the mean time to death in murine models of infection [27–30]. These data suggest *B*. *pseudomallei* and *B*. *mallei* polysaccharides are immunologically important and potentially viable vaccine candidates.

Given that *B*. *pseudomallei* and *B*. *mallei* are facultative intracellular pathogens, an antibody response alone in melioidosis and glanders patients might not be sufficient in controlling and eliminating the infection. A common drawback of many of the current vaccine approaches is their lack of ability to stimulate cell-mediated immunity [31]. The protective efficacy of live-attenuated *B*. *pseudomallei* has been evaluated in murine models of melioidosis using multiple routes of immunizations; these have provided partial protection against challenge with virulent *B*. *pseudomallei* [32–35]. However, the ability of this live attenuated strain to potentially establish latent infections in immunocompromised individuals will likely preclude the use of such attenuated *Burkholderia* strains as vaccines in humans.

*Salmonella*-based live vector vaccines have been extensively developed and used for delivering foreign antigens to the immune system against a number of pathogens [36–38]. Attenuated *Salmonella* strains were reported to strongly trigger innate immune responses, as they express LPS and flagella and contain stimulatory CpG motifs that stimulate Toll-like receptors. In addition, these strains also induce strong Th1-type responses in mice [39,40] and humans [41,42].

The identification of the biosynthetic gene cluster responsible for addition of O antigen onto the lipid A-core of *B*. *mallei* was originally described by Burtnick *et al*. [14]. Here, we transferred the *B*. *mallei* O antigen synthesis locus, which resides on the plasmid p1C3, to the attenuated *aroA* mutant of *Salmonella enterica* serovar Typhimurium, SL3261. This construct combined the adjuvant properties and advantages of the live-attenuated *Salmonella* strain as a delivery vehicle for the *B*. *mallei* O-antigen. LPS isolated from the recombinant strain, SL3261/p1C3 was reactive with monoclonal antibodies specific to *B*. *mallei* O antigen. This attenuated strain was delivered intranasally and evaluated for its protective efficacy in mice against lethal intranasal infection with *B*. *thailandensis* as a model of melioidosis and glanders.

## Materials and Methods

### Ethics Statement

Use of animals in this study was reviewed and approved by the University of Virginia Institutional Animal Care and Use Committee (IACUC) under protocol number 2844-02-11. All mice were kept under specific pathogen-free conditions, and all guidelines for humane endpoints were strictly followed. All animal experiments were conducted in accordance with the “Public Health Service Policy on Humane Care and Use of Laboratory Animals” by NIH, “Animal Welfare Act and Amendments” by USDA, “Guide for the Care and Use of Laboratory Animals” by National Research Council (NRC).

### Antibodies

5C8-1C3, an anti-*B*. *mallei* O antigen mouse monoclonal IgG antibody and 3B3-5, an anti-*B*. *pseudomallei* O antigen mouse monoclonal IgG antibody were used in Western blots and ELISA. Pp-PS-W, a mouse monoclonal IgM antibody specific to O-PS-II, a CPS of *B*. *pseudomallei*, was used in ELISA (kindly supplied by Dr. Paul Brett (University of South Alabama) [43]).

### Transduction of *B*. *mallei* O antigen biosynthetic locus into *Salmonella enterica* serovar Typhimurium strain SL3261

The identification of the biosynthetic gene cluster responsible for the addition of O antigen onto the lipid A-core of *B*. *mallei* was originally described by Burtnick *et al*. [14]. This locus is contained on plasmid p1C3 (generously supplied by Dr. Donald Woods (University of Calgary)). The plasmid was introduced into the attenuated *aroA* mutant *S*. *enterica* serovar Typhimurium strain SL3261 [44] via transduction, as previously described [45], resulting in the recombinant vaccine strain SL3261/p1C3.

### Preparation of bacterial strains for vaccination and infection

*Salmonella enterica* serovar Typhimurium strain SL3261, containing the plasmid p1C3 expressing *B*. *mallei* O antigen (vaccine), or SL3261 not containing the plasmid (vector), were used for immunization. All strains were grown overnight in Luria broth (LB) or LB supplemented with 100 μg/ml ampicillin, respectively. Both strains were subcultured and grown to an OD_650_ of 0.5. Bacteria were then washed twice and resuspended in phosphate-buffered saline (PBS), pH 7.4, and adjusted spectrophotometrically to obtain the desired immunization dose. For infection, *B*. *thailandensis* E264 [46] was grown in trypticase soy broth (TSB) overnight, sub-cultured, and grown to an OD_600_ of 0.5. Cells were harvested by centrifugation and washed twice in PBS. Before administration to animals, bacterial cells were adjusted spectrophotometrically to obtain the desired density.

### *B*. *mallei* and *B*. *pseudomallei* LPS extraction

LB inoculated with *B*. *mallei* (ATCC 23344) or *B*. *pseudomallei* (ATCC 23343) was incubated overnight with vigorous shaking. Cell pellets were obtained by centrifugation and LPS was extracted using a modified hot aqueous-phenol procedure [47]. Following extraction, the resulting phenol and aqueous phases were combined and dialyzed in distilled water to remove the phenol. The dialysates were then clarified by centrifugation and concentrated by lyophilization. The crude preparations were solubilized to a concentration of 20 mg/ml in RD buffer (10 mM Tris-HCl pH 7.5, 1 mM MgCl_2_, 1 mM CaCl_2_), and 2 μg/ml of DNase I from bovine pancreas was added followed by incubation at 37°C for 2 h. RNase A was then added to the mixture (2 μg/ml) and the incubation continued for 2 h. Proteinase K was then added to a final concentration of 20 μg/ml and the mixture was incubated for at least 3 h at 50°C. The samples were clarified by centrifugation and LPS was isolated from the supernatants as precipitated gels following repeated ultracentrifugation at 100,000 X *g* and 4°C until the A_260_ and A_280_ were less than 0.02. After the final spin, the gelatinous pellets were resuspended in pyrogen-free water and lyophilized. To remove contaminating phospholipids, lyophilized LPS samples were repeatedly extracted with 90% ethanol.

### Sodium dodecyl sulfate-polyacrylamide gel electrophoresis (SDS-PAGE) and immunoblotting

To confirm the expression of *B*. *mallei* O-antigen, SDS-PAGE and Western blot analyses were performed as previously described [48]. Whole-cell lysates of *Salmonella* organisms were separated SDS-PAGE and analyzed by Western blotting. As controls, LPS purified from *B*. *mallei* and *B*. *pseudomallei* were used.

### Intranasal vaccination and challenge

Four- to six- week old female BALB/c mice were obtained from Harlan Sprague-Dawley Farms (Chicago, IL). The University of Virginia Animal Care and Use Committee approved all procedures concerning the use of mice for this study. Mice were housed at the University of Virginia under SPF conditions, and were fed *ad libitum*; mice were given one week of acclimation before the experiments were initiated. BALB/c mice were anesthetized intraperitoneally with 200 μl of ketamine (6.7 mg/ml) and xylazine (1.3 mg/ml) in a 0.9% saline solution prior to vaccination. Mice were intranasally instilled with 20 μl (10 μl per nostril) of PBS, 10^7^ colony forming units (CFU) SL3261, or 10^7^ CFU SL3261/p1C3. Boosters were performed using the same protocol as the initial vaccination, approximately 14 days after the initial vaccination. Blood was collected via the lateral tail vein at four weeks post-immunization. The serum was separated and used for detection of antigen-specific antibodies by enzyme-linked immunosorbent assay (ELISA). *B*. *thailandensis* E264 was used for all infection studies in immunized mice. The LD_50_ was determined according to the method of Reed and Muench [49] and calculated to be 1 x 10^6^ CFU. Six weeks post initial vaccination, mice were anesthetized as described above and 5 x 10^6^ CFU of *B*. *thailandensis* (5 x LD_50_) were given in a 20 μl volume (10 μl per nostril). Mice were closely monitored and checked every six hours for signs of morbidity up to 150 hours during the course of the infection. Mice that became morbid with ruffled fur, shaking, unresponsive to touch, unable to move, and unable to obtain food and water were humanely euthanized by CO_2_.

### Indirect ELISA

Immulon 2 HB ELISA plates (Thermo Labsystems, Franklin, Mass.) were coated with 100 μl of either 10 μg/ml *B*. *mallei* LPS, or 10 μg/ml of *B*. *pseudomallei* LPS (as extracted above) in PBS supplemented with 20 mM MgCl_2_ pH 7.2 for 1 h at 37°C or with ~5 x 10^7^ CFU/well of *B*. *thailandensis* E264 overnight at 4°C. Following a 1 h blocking step with 5% skim milk / PBS-0.05% Tween-20 (PBS-T) at 37°C. Serum samples were serially diluted in PBS- 2% bovine serum albumin (PBS-B) and 100 μl were placed into each well in either *B*. *mallei* LPS-coated, *B*. *pseudomallei* LPS-coated, or *B*. *thailandensis*-coated plates, in duplicate. Mouse monoclonal antibodies (mAb) 5C8-1C3, 3B3-5, and Pp-PS-W were used as positive controls. After overnight incubation at 4°C, the plates were washed three times with PBS-T and air-dried. Secondary antibodies (anti-mouse total IgG, IgG1, IgG2a, IgG2b, IgG3, or IgM conjugated to alkaline phosphatase (Southern Biotechnology Associates, Inc., Birmingham, AL)) were added to individual plates, diluted 1:5000 in PBS-B, and incubated at 37°C for 1 h. The microwells were washed again, and the enzymatic reaction was developed by the addition of 4-nitrophenol phosphate disodium salt hexahydrate (PNPP) diluted to 1 mg/ml in PNPP substrate solution (10% diethanolamine, 25 μM MgCl_2_). The reaction was terminated by the addition of 3 M NaOH to each well, and the results were recorded by using a Thermo microplate reader (Molecular Devices, CA) measuring the colorimetric reaction at OD_405_ with the data was displayed using SOFTmax Pro version 1.1 software.

### Detection of bacterial load

Prior to sample collection, mice were euthanized by CO_2_ asphyxiation, after which bacterial loads were quantified by collecting the nasal wash (NW) and harvesting organs. For the NW, an 18-G catheter was placed at the oropharyngeal opening of the mouse and 0.5 ml of PBS-B was flushed through the nasal passage and collected. Lungs, livers, and spleens were aseptically removed from mice, weighed, and homogenized in 1 ml PBS-B. The CFU of *B*. *thailandensis* E264 were determined by plating 10-fold serial dilutions of the homogenates on tryptic soy agar (TSA) and Ashdown’s agar plates, which is a selective culture medium for the isolation and characterization of *Burkholderia spp*. Final results were expressed as CFU/ml for NW and CFU/g for organs.

### Histopathology

24 and 72 h post-infection with 5 x 10^6^ CFU *B*. *thailandensis* (5 x LD_50_), lungs of BALB/c mice were isolated in their entirety. Under sterile conditions, the trachea of each animal was exposed and the lungs were inflated with 0.3 ml of 10% neutral-buffered formalin, removed and immediately immersed in the same fixative. Livers and spleens were also used for histology; these organs were collected under the same conditions as the lungs. All samples were processed by standard paraffin embedding methods. Sections were cut 2 μm thick and stained with haematoxylin-eosin (H&E). The preparation of tissue sections was performed by the University of Virginia Research Histology Core Facility. Tissue sections were examined by a veterinary pathologist who was blinded to animal group assignments.

### Cytokine analysis

The concentrations of the selected cytokines from pooled sera collected from PBS-, vector-, and vaccine-immunized mice were measured in duplicate using the Invitrogen Mouse Cytokine Magnetic 20-Plex Panel (Invitrogen) according to the manufacturer’s protocol. Samples were acquired using the Luminex 200 analyzer system (Luminex Corporation, USA), and the data were analyzed using xPONENT version 3.1 software (Lifetechnologies, Carlsbad, CA).

### Opsonophagocytosis assay

Luminescent opsonophagocytosis assay was performed according to the method previously described by DiGiandomenico *et al*. [50]. In this assay, luminescent *B*. *thailandensis* E264 was engineered to be luminescent, as previously described [51], where luminescence is only observed with accessible ATP indicating live bacteria. Briefly, the assay was performed in 96-well plates using 25 μl of each OPK component: *B*. *thailandensis* strain, E264*lux*, from log-phase cultures diluted to 2 x 10^6^ CFU/ml; diluted baby rabbit serum (1:10); 2 x10^7^ polymorphonuclear leukocyte (PMN); and sera collected from PBS-, vector-, or vaccine-immunized mice. The percentage of killing was determined by comparing the relative luciferase units (RLU) derived from assays lacking serum to the RLU obtained from assays with vector or vaccine sera following a 120 min incubation at 37°C shaking at 250 RPM. Microtiter plates were read using an Envision Multilabel plate reader (PerkinElmer).

### Statistical analysis

All analyses were performed using GraphPad Prism version 4 software. ELISA endpoint titers were calculated using the linear regression of duplicate measurements of adjusted OD_405_ and were expressed as the reciprocal dilution. The *x-* intercept served as the endpoint titer. Antibody titers were compared using the Kruskal-Wallis test for comparison of three groups or the Mann-Whitney *U* test for two group analysis. The results of survival studies were represented using Kaplan-Meier survival curves and were analyzed by the log-rank test.

## Results

### Expression of *B*. *mallei* O antigen

**Fig 1:**
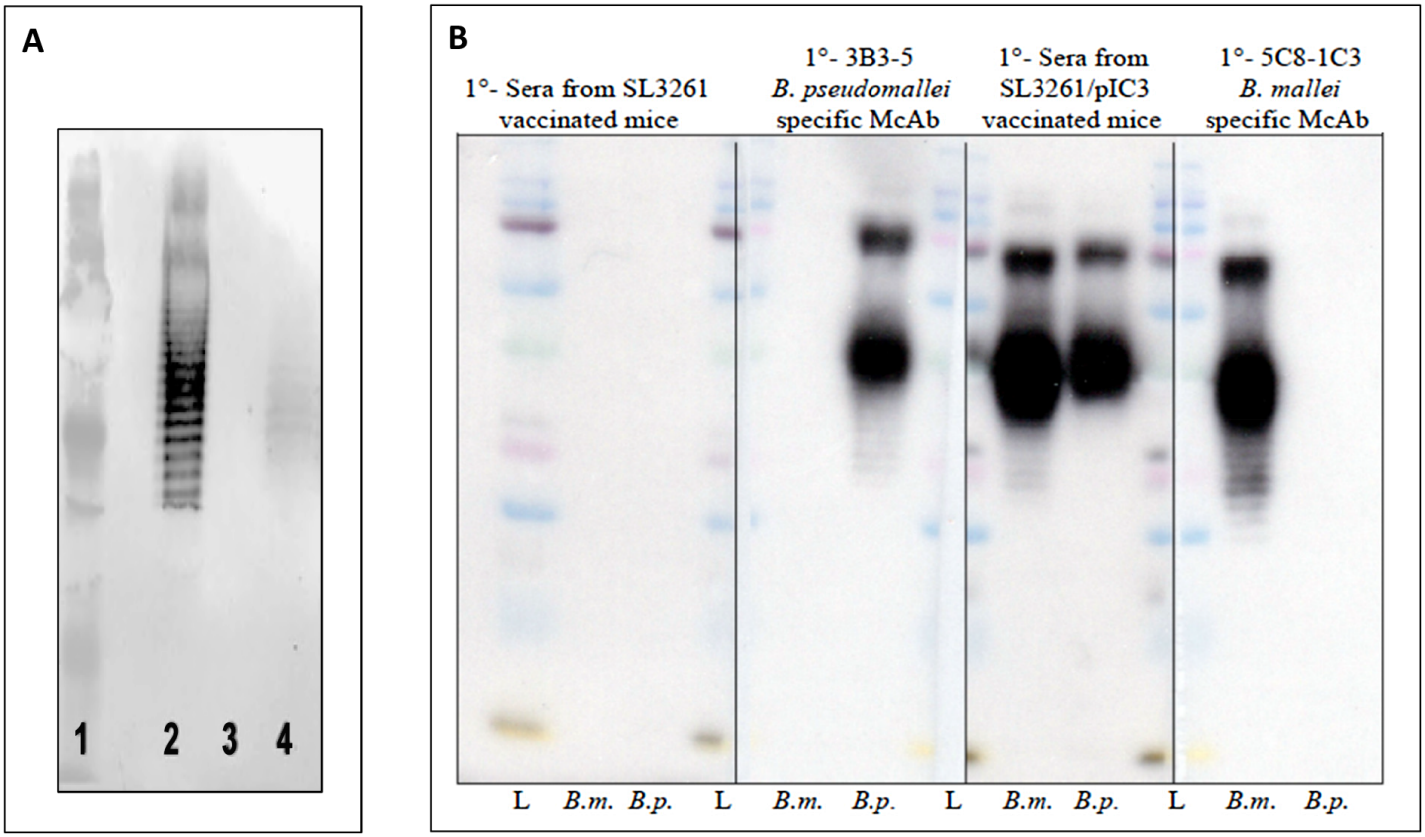
Analysis of LPS expression and immunoreactivity. **(A)** LPS extracted from *B*. *mallei* (Lane 2), SL3261 (Lane 3), and SL3261/p1C3 (Lane 4) was subjected to SDS-PAGE followed by immunoblotting analysis using 5C8-1C3 anti-*B*. *mallei*-specific LPS monoclonal antibody (mAb). Molecular weight marker is shown in Lane 1. **(B)** LPS extracted from *B*. *mallei* (*B*.*m*.) and *B*. *pseudomallei* (*B*.*p*) was run, subjected to SDS-PAGE followed by immunoblot using sera collected from SL3261 (vector)-immunized mice; 3B3-5, anti-*B*. *pseudomallei* mAb; sera collected from SL3261/p1C3 (vaccine)-immunized mice; and 5C8-1C3, anti-*B*. *mallei* mAb. Molecular weight ladder (L).

Plasmid p1C3, which contains the genes that encode proteins for production of the *B*. *mallei* O antigen, was transferred to the live-attenuated vaccine strain *Salmonella enterica* serovar Typhimurium SL3261. In order to determine whether SL3261 was capable of expressing the *B*. *mallei* O antigen, LPS was extracted from strains SL3261 and SL3261/p1C3 and analyzed by immunoblotting with a monoclonal antibody (mAb) specific for the *B*. *mallei* O antigen (Fig 1A). Our results indicate that SL3261/pIC3 is capable of expressing the *B*. *mallei* O-antigen (Fig 1A).

### SL3261/p1C3 induces serum antibodies to *B*. *mallei*, *B*. *pseudomallei*, and *B*. *thailandensis* after intranasal immunization

To determine whether heterologously expressed *B*. *mallei* O antigen was immune accessible, pooled sera from intranasally immunized mice were analyzed by Western immunoblot using LPS extracted from *B*. *mallei* and *B*. *pseudomallei*. Sera collected from vaccine-immunized mice reacted with LPS of both *B*. *mallei* and *B*. *pseudomallei*. No reactivity was observed with mouse antisera from vector-immunized mice (Fig 1B). As controls, purified *B*. *mallei* and *B*. *pseudomallei* LPS were probed with 5C8-1C3 and 3B3-5 mAbs specific to LPS of *B*. *mallei* and *B*. *pseudomallei*, respectively. As anticipated, we did not observe any cross-reaction between the LPS of *B*. *mallei* and 3B3-5, the anti-*B*. *pseudomallei* or between LPS extracted from *B*. *pseudomallei* and 5C8-1C3, the anti-*B*. *mallei* mAb (Fig 1B).

**Fig 2:**
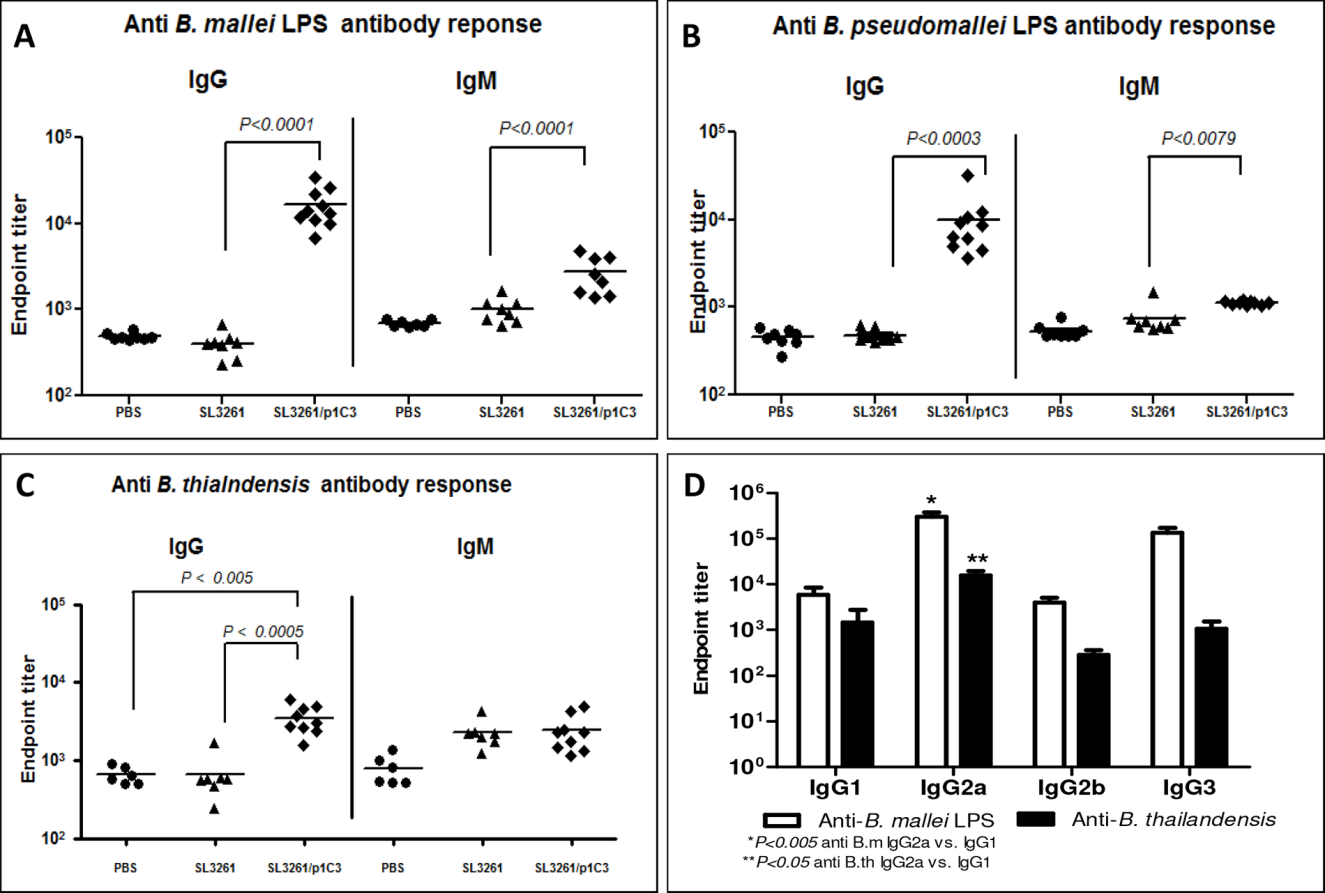
Serum antibody response of BALB/c mice following intranasal immunization with *S*. *enterica* serovar Typhimurium SL3261 expressing *B*. *mallei* O antigen. Sera were collected 4 weeks post-vaccination, and the data were analyzed by the Mann-Whitney *U* test. Serum IgG and IgM response to: **(A)** *B*. *mallei* LPS (*P* < 0.0001 vector vs. vaccine for IgG and IgM); **(B)** *B*. *pseudomallei* LPS (*P* < 0.0003 vector vs. vaccine for IgG, and *P* < 0.0079 for IgM); **(C)** *B*. *thailandensis* E264 lysate (*P* < 0.005 PBS vs. vaccine, and *P* < 0.0005 vector vs. vaccine). **(D)** Specific IgG response in the sera of intranasally-immunized BALB/c mice to *B*. *mallei* LPS or anti-*B*. *thailandensis*.

ELISA analysis using sera collected four weeks post-immunization from BALB/c mice that received the vaccine revealed robust *B*. *mallei* LPS-specific IgG and IgM antibody response when compared with sera from the vector-immunized or PBS control mice (Fig 2A). Moreover, sera from intranasally vaccinated animals were also reactive to *B*. *pseudomallei* LPS (Fig 2B). We next examined the IgG and IgM antibodies specific to *B*. *thailandensis*. Sera from vaccine-immunized BALB/c mice after intranasal immunization showed significant levels of anti-*B*. *thailandensis* IgG compared to the control groups. *B*. *thailandensis*-specific IgM levels in the vaccine group were not significantly greater when compared to those of the vector-immunized groups (Fig 2C).

The serum IgG subtype responses to *B*. *mallei* LPS and *B*. *thailandensis* were also determined for vaccine-immunized animals. Intranasal immunization elicited significantly higher levels of IgG2a than IgG1 and IgG3 (*P<0*.*005* anti-*B*. *mallei* LPS IgG2a vs. IgG1, and *P<0*.*05* anti-*B*. *thailandensis* IgG2a vs. IgG1). We also observed distinct preferences of IgG subclasses to *B*. *mallei* LPS (IgG2a> IgG1>IgG3>IgG3b) and B. thailandensis (IgG2a> IgG3>IgG1>IgG2b). A determination of the IgG1 and IgG2a isotypes ratio in the serum of vaccine-immunized mice was also performed as a surrogate for Th1 and Th2 responses. Interestingly, immunization with SL3261/p1C3 resulted in low IgG1/IgG2a ratios of 0.1 and 0.4 for reactivity with *B*. *mallei* LPS and *B*. *thailandensis*, respectively, indicative of a predominantly Th1 response.

### Cytokine response to vaccination with *Salmonella enterica* serovar Typhimurium, SL3261

**Fig 3:**
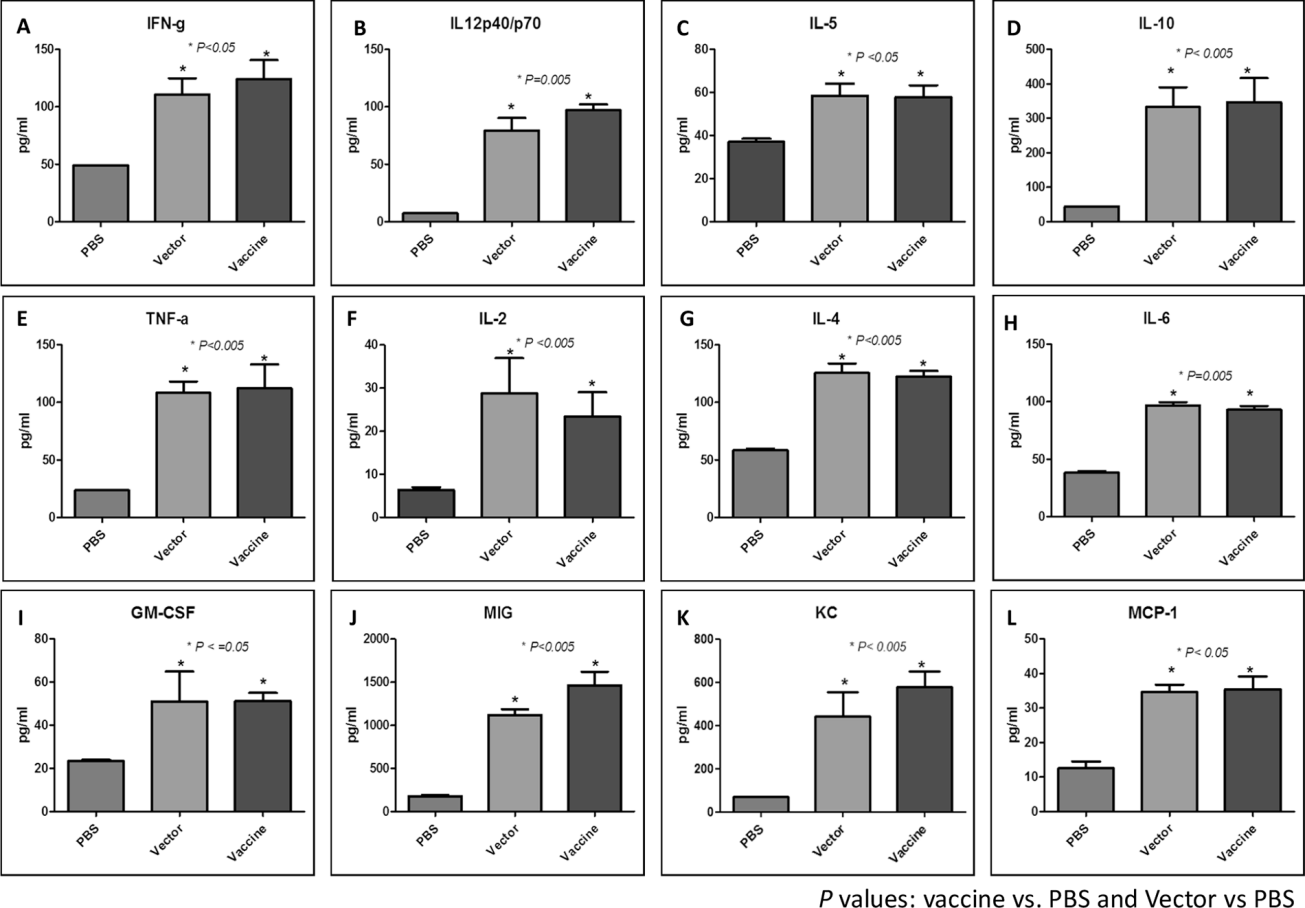
Serum cytokine response of BALB/c mice following intranasal immunization with *S*. *enterica* serovar Typhimurium SL3261 expressing *B*. *mallei* O antigen. Sera were collected 4 weeks post-vaccination, and the data were analyzed by the Mann-Whitney *U* test. *P* values were calculated as: Vaccine vs. PBS and Vector vs. PBS.

The cytokine/chemokine responses of the PBS, vector-, and vaccine-immunized mice at 4 weeks post-initial immunization were determined by quantification of a panel of Th1/Th2 cytokines in pooled sera using a mouse cytokine magnetic 20-Plex Panel. Significantly higher levels of IFN-γ, IL-12p70, IL-5, TNF-α, and IL-10 were detected in the pooled sera of vaccine and vector immunized animals when compared to the respective PBS immunized mice. Also greater serum concentrations of IL-2, IL-4, IL-6, and GM-CSF were observed in vaccine- and vector-immunized mice (Fig 3).

### Vaccine-mediated protection against lethal challenge with *B*. *thailandensis* E264

**Fig 4:**
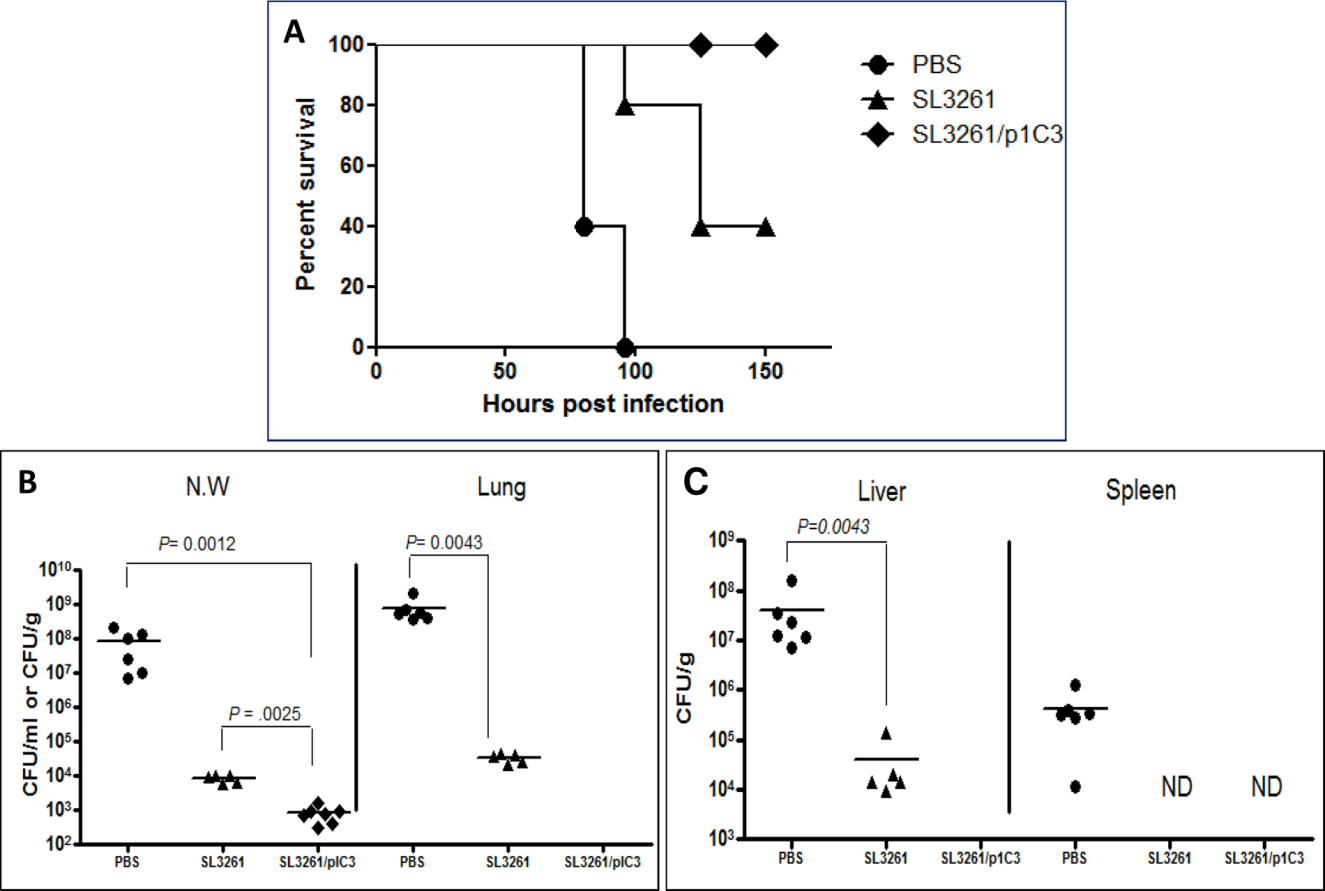
Survival rates and bacterial loads in organs of intranasally immunized BALB/c mice after intranasal challenge with *B*. *thailandensis* (5 x 10^6^ CFU). **(A)** Mice were immunized with PBS, SL3261 (vector), or SL3261/p1C3 (vaccine) via the intranasal route, then challenged. Mice were monitored for survival for a period up 150 h post-challenge. Results are represented in Kaplan-Meier survival curves and were analyzed by log-rank test. Log-rank: PBS vs. vaccine (*P =* 0.0031); vector vs. vaccine (*P =* 0.0128). Median survival: PBS, 64 h; SL3261, 84 h; and SL3261/p1C3, undefined). Results are representative of three independent experiments, five mice each. **(B, C)** Bacterial load in the organs of intranasally immunized mice 72 h post-challenge. All samples were plated for viable CFU on TSA and Ashdown’s agar. Each point represents a single mouse. ND indicates not detected. Data was analyzed by the Mann-Whitney *U* test. Nasal wash (*P* = 0.0012 vaccine vs. vector and *P* = 0.0025 vaccine vs. PBS); lung (*P* = 0.0043 vector vs. PBS); liver (*P* = 0.0043 vector vs. PBS).

To investigate the ability of the SL3261/p1C3 to confer protection, vaccinated animals were intranasally challenged with a lethal dose of *B*. *thailandensis* (5 x 10^6^ CFU). No morbidity was observed in vaccine-immunized mice. On the other hand, all PBS-treated mice were dead at 100 h post-infection and 60% of vector-immunized mice were dead at 125 h post-infection (Fig 4A).

To further determine if intranasal vaccination promoted the clearance and/or limited the dissemination of *B*. *thailandensis*, the bacterial burden in various organs was determined at 72 h post-infection in PBS-, vector-, and vaccine-immunized groups. The nasal wash, lung, spleen, and liver from each mouse were collected and the viable bacterial count in each organ was determined. At 72 h post-challenge, NW from the vaccine-immunized animals had fewer bacterial CFU compared to vector- and PBS-immunized mice (Fig 4B). Vaccine-immunized mice were more capable of clearing *B*. *thailandensis*, as no bacteria were detected in any of the examined organs of these mice compared to the control-treated mice. Interestingly, vector-immunized mice showed some ability to control the infection; the bacterial CFU recovered from their NW, lung, and liver were significantly lower than those recovered from PBS-treated mice Fig 4B and 4C.

### Histopathological changes in response to intranasal challenge with *B*. *thailandensis*

**Fig 5:**
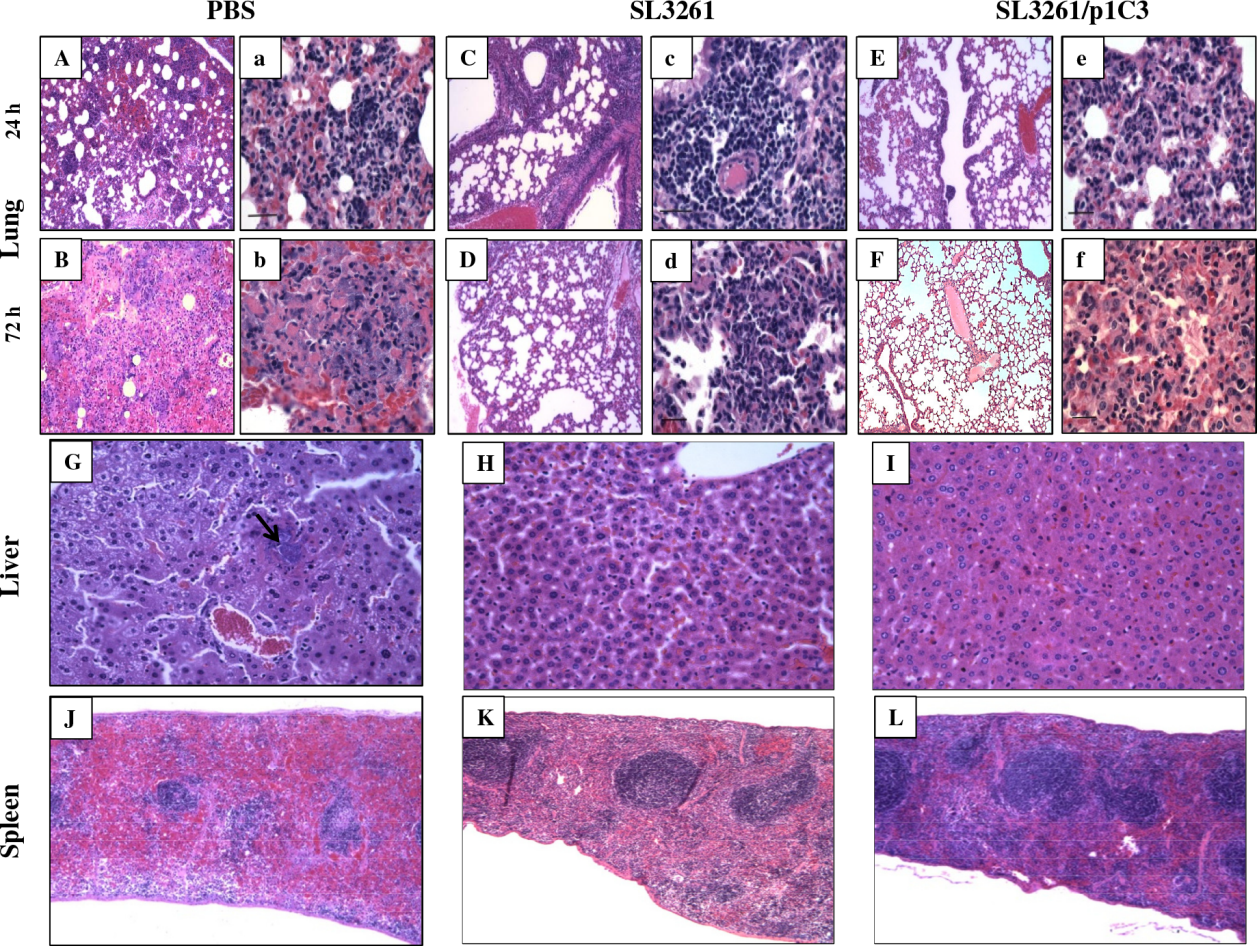
Histopathological changes in response to intranasal challenge with *B*. *thailandensis* E264. Lungs of vaccinated BALB/c mice were isolated in their entirety at 24 **(A-C)** and 72 h **(D-F)** post-infection with 5 x 10^6^ CFU *B*. *thailandensis* (5 x LD_50_). The tracheas of each animal were exposed and inflated with 0.3 ml of 10% neutral-buffered formalin and immediately immersed in the same fixative. Livers **(G-I)** and spleens **(K-L)** were also used for histology; organs were collected under the same conditions. All samples were processed by standard paraffin embedding methods; sections were cut 2 mM thick and stained with haematoxylin-eosin (H & E). Preparation of tissue sections was performed by the University of Virginia Research Histology Core Facility. Tissue sections were examined by a veterinary pathologist who was blinded to animal group assignments. Sections A, B, C, D, E, and F are shown in magnification, 10x; representative sections a, b, c, d, e, and f are in magnification, 40x. Liver and spleen sections are shown in magnifications 10x and 5x, respectively.

The histopathological changes in the lungs, livers, and spleens of PBS-, vector-, and vaccine-immunized mice following challenge with *B*. *thailandenis* at 24 and 72 h post-infection were assessed in an independent experiment. We noted extensive signs of inflammation in the lung sections of PBS mice that succumbed to intranasal challenge with *B*. *thailandensis*. At 24 h, lung sections from this group displayed multifocal areas of hemorrhage intermixed with areas of mild to moderate infiltration of neutrophils Fig 5A and 5a. By 72 h, lung sections from the same group displayed multifocal to diffuse hemorrhage, necrosis and edema intermixed with multifocal areas of severe infiltration of neutrophils, and focal aggregates of hemosiderin-laden macrophages. The bronchi showed moderate infiltration of neutrophils often admixed with large numbers of bacterial colonies Fig 5B and 5b. Lung sections from vector-immunized animals at 24 h displayed signs of congestion, multifocal areas of hemorrhage, and moderate infiltration of neutrophils and macrophages. Multifocal peri-bronchial infiltration of moderate numbers of plasma cells and lymphocytes were also noted Fig 5C and 5c. At 72 h post-infection with *B*. *thailandensis*, the alveolar walls were thickened by Type II cell hyperplasia and infiltration of moderate numbers of neutrophils Fig 5D and 5d. At 24 h post-infection, lung sections of vaccine-immunized mice displayed multifocal areas of hemorrhage. The alveolar walls are diffusely thickened by type II cell hyperplasia Fig 5E and 5e. While at 72 h, infiltration of lymphocytes and plasma cells multifocal areas of severe infiltration of foamy macrophages, neutrophils, and hemosiderin-laden macrophages were noted in the peri- bronchial and parenchyma in lung sections of this group Fig 5F and 5f.

Liver sections from PBS-immunized mice showed multifocal areas of necrosis infiltrated by moderate numbers of degenerate neutrophils surrounding bacterial colonies at 72 h post-infection (Fig 5G). Mild infiltration of lymphocytes, with multifocal areas of necrosis infiltrated by moderate numbers of degenerate neutrophils was observed in liver section of vector-immunized mice (Fig 5H). On the other hand, liver sections from vaccine-immunized mice showed occasional portal areas with mild infiltration of lymphocytes (Fig 5I). Spleen sections from PBS-treated mice displayed significant lymphoid depletion, collapse of sinusoidal spaces at 72 h post infection (Fig 5J). In contrast, while spleen sections from vector-immunized mice showed diffuse moderate lymphoid depletion, no histopathological changes were observed in spleen sections of vaccine-immunized mice Fig 5K and 5L.

### Antibody response induced by intranasal vaccination mediate efficient opsonic killing of *B*. *thailandensis in vitro*

**Fig 6:**
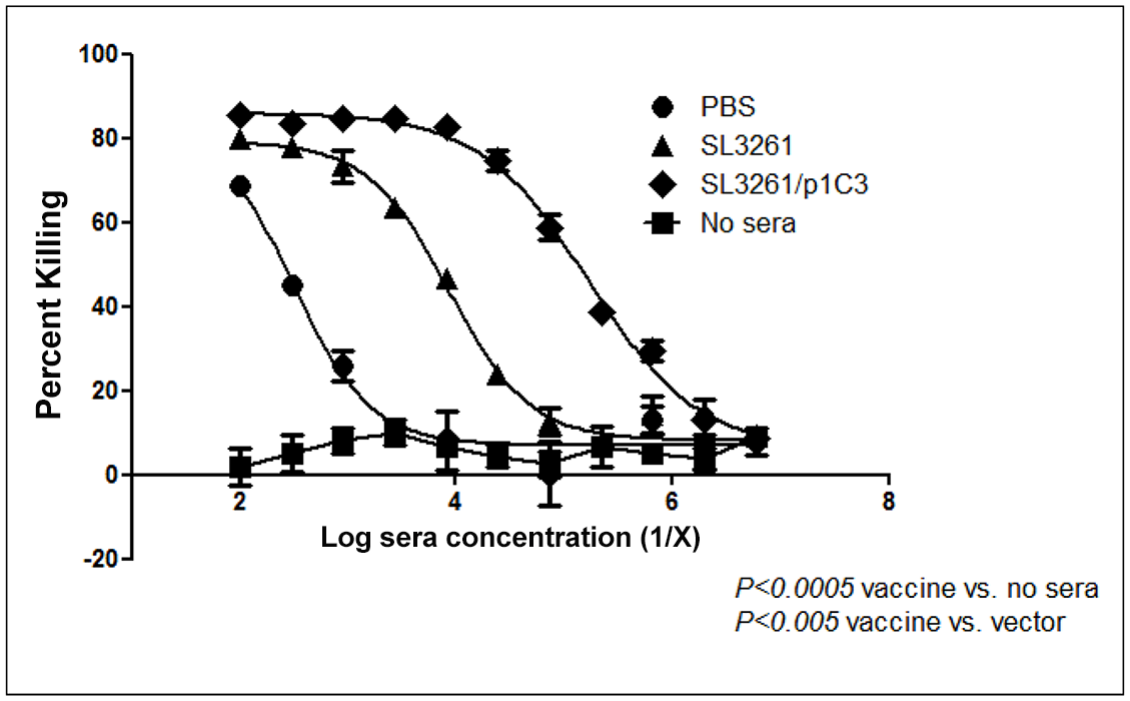
Opsonophagocytosis killing of *B*. *thailandensis*. *In vitro* opsonophagocytic killing of *B*. *thailandensis* E264*lux* using dilutions of pooled antisera collected from intranasally PBS-, SL3261 (vector)-, and SL3261/p1C3 (vaccine)-immunized BALB/c mice. Plates were read at 120 min following the co-incubation of the opsonophagocytosis assay components. Bars represent mean percentage of killing, and error bars represent the standard deviation. Data were analyzed by the Mann-Whitney *U* test (*P<0*.*0005* vaccine vs. no sera; *P<0*.*005* vaccine vs. vector).

We examined the efficiency immune sera to promote opsonization and phagocytosis of *B*. *thailandensis* by PMN. As a control, we performed the assay in absence of vaccine-immune sera to further confirm the role of antibodies in the protection. Pooled antisera from vaccine-immunized mice mediated biologically significant level of opsonophagocytic killing (>50%) of *B*. *thailandensis* strains at serum dilutions up to 1:72,900 compared to limited killing observed with pooled sera from vector-immunized mice (1:2,700). Furthermore, no killing of strain E264*lux* was observed in absence of vaccine immune sera (Fig 6). These results suggest that one of the mechanisms for the observed protection is opsonophagocytosis of *B*. *thailandensis*.

## Discussion

Given the worldwide emerging infectious disease problem, bioterrorism threat, and increasing incidence of antibiotic resistance, more effective measures for prevention and treatment of *B*. *pseudomallei* and *B*. *mallei* infections are urgently needed. In the present work, we demonstrate that intranasal immunization of mice with attenuated *S*. *enterica* serovar Typhimurium strain SL3261 expressing *B*. *mallei* O antigen results in the development of a protective immune response against subsequent lethal challenge with *B*. *thailandensis* E264. We have used SL3261 as a surrogate for the safe human vaccine strain, *Salmonella enterica* subsp. *enterica* serovar Typhi (*S*. *typhi*) Ty21a, which is given orally to protect against typhoid fever [52]. Ty21a vaccine has been proposed as vehicles to deliver heterologous antigens. Unfortunately, pre-clinical studies with Ty21a are hampered because mice are generally resistant to oral infection with this human-specific pathogen [53,54]. More recently intranasal administration has been used as a route to mimic the mucosal infection and immunization with *S*. *typhi* [55–57].

We have previously had success using *Salmonella*-based live vector vaccines to express O antigen of *Pseudomonas aeruginosa* [45] and have observed that intranasal immunization is provides superior protection compared to oral immunization [48,58]. We have shown that intranasal immunization of mice with attenuated *Salmonella* heterologously expressing *P*. *aeruginosa* O antigen via multiple routes induced protective immune responses against lethal infection with *P*. *aeruginosa* in several mouse models [58]. Similar experiments will be needed with the *B*. *mallei* LPS-based vaccine described here to determine which route of immunization provides optimal protection and also whether we observe similar results when this antigen is expressed in *S*. *typhi* Ty21a.

Immunization with purified bacterial polysaccharides predominantly generates short-lived and low-affinity antibody responses predominated with IgM and IgG3 [27]. It has been previously shown that conjugation of the bacterial polysaccharide to a carrier protein induces an immunogenic response to conjugated carbohydrates as a result of the switch from a generally T-cell independent to a T-cell dependent response (reviewed in [59]).

Our selection of O antigen as a vaccine candidate was based on findings that LPS expressed by *B*. *mallei* and *B*. *pseudomallei* is both a virulence determinant and a protective antigen [60,61]. In addition, previous studies have demonstrated that the LPS expressed by *B*. *pseudomallei* and *B*. *thailandensis* are structurally similar [16,19]. The O-polysaccharide moieties are composed of unbranched heteropolymers consisting of disaccharide repeats having the structure -3-β-D-glucopyranose-(1–3)-6-deoxy-α-L-talopyranose [14,43,62]. In addition, it was also shown that *B*. *mallei* expresses O antigens that are structurally similar to those expressed by *B*. *pseudomallei* and *B*. *thailandensis* with the exception of the L-6dTal*p* residues lacking acetyl modifications at the *O*-4 position due to the absence of *oacA* gene in *B*. *mallei* [14,63]. Although four different phenotypes of *B*. *pseudomallei* LPS O antigen have been recently described [19], most *B*. *pseudomallei* species express type A O antigen, which is considered the backbone and most abundant structure among *Burkholderia* species [19,64]. Most importantly, the association between LPS heterogeneity in *B*. *pseudomallei* and disease severity, clinical manifestation or underlying risk factors have not been reported [65]. From the standpoint of vaccine development, the structural similarity that exists between the characterized LPS of these species suggests the potential of a single vaccine against both pathogens. Consistent with the minimal structural difference between the LPS of these species, we found that the serum antibody response generated following intranasal immunization with the recombinant vaccine, but not the vector, reacted with the LPS of *B*. *pseudomallei* and *B*. *mallei* Figs 1B, 2A and 2B. Qazi *et al*. have previously highlighted the antigenic similarities between the LPS isolated from *B*. *thailandensis*, *B*. *pseudomallei*, and *B*. *mallei* using serum from animals immunized with either heat-killed *B*. *mallei* 23344 or *B*. *pseudomallei* K96243 [16]. Consistent with this observation, our results demonstrated that sera from vaccine-immunized mice also reacted with *B*. *thailandensis* (Fig 2C).

The presence of *B*. *mallei* LPS- and *B*. *thailandensis*-specific IgG1 and IgG2a antibodies in serum of the vaccinated mice suggest that the recombinant *Salmonella* expressing *B*. *mallei* O antigen vaccine induced a mix of Th1 and Th2 immune responses. Vaccination appeared to induce a higher proportion of IgG2a antibodies to *B*. *mallei* LPS and *B*. *thailandensis*.

In general, a Th1 type of immune responses is considered desirable for protection against intracellular bacterial infections, such as *Burkholderia*. Amemiya *et al*. [66] have previously reported that a Th2 antibody response dominated with IgG1 was not adequate in protecting against lethal challenge with *B*. *mallei* ATCC 23344 and suggested that a decrease in the IgG1/IgG2a ratio might result in enhanced protection. We assessed the relation of levels of IgG1 to IgG2a as a surrogate marker for the Th1 and Th2 balance, the IgG1/IgG2a ratio was <1 suggesting a shift towards a Th1-type response. Moreover, analysis of the Th1/Th2 cytokines and chemokines panel indicated that vaccine and vector immunized animals exhibited greater serum concentrations of all cytokines in comparison to PBS-treated mice. The presence of IFN-γ or TNF-α is consistent with a Th1-skewed immune response (Fig 3). The induction of a strong Th1 response following immunization with *Salmonella* vaccine strains has been reported in animal models [39,40] and in humans [41,42]. Consistent with these findings, our data indicates that immunization with SL3261/p1C3 generated a mixture of Th1 and Th2 immune response. Nevertheless, the significant increase of IFN-γ, IL-12, and TNF-α suggests a Th1 skewed response.

The histopathological changes in the lungs, spleens, and livers from PBS-treated mice indicated a notable inflammatory response, which is consistent with bacteremia (Fig 5). In contrast, vector- and vaccine-immunized mice demonstrated moderate to minimal pulmonary signs of inflammation, respectively. The increased levels of cytokines and chemokines observed in the pooled sera obtained from vector- and vaccine-immunized mice paralleled the corresponding ability of these mice to resist the challenge with *B*. *thailandensis*. Our findings are consistent with recent observations by Morici *et al*. [20] and West *et al*. [23] who observed similar responses to *B*. *thailandensis* in susceptible BALB/c mice. Other groups have demonstrated that *Salmonella* strains induce strong innate and acquired immunity in animal models through the induction of a cytokine storm including TNF-α, IFN-γ, IL-6, IL-12, IL-18, and variety of chemokines that activates and recruits immune cells including neutrophils and macrophages to the site of infection in response to LPS, flagella, stimulatory CpG motifs and their cell wall components [39,67,68]. It was previously reported that the IL-12 burst following infection with *Salmonella* serovar Typhimurium promotes Th1 immune response that contribute to the elimination of the bacteria through IFN-γ production [69]. Likewise, our data indicate that vector-immunized mice were better equipped to control the *B*. *thailandensis* infection when compared to the PBS-treated group, however these vector-immunized mice were still not protected from death. These results support the hypothesis that a Th1-driven immune response is necessary for optimal protection. At this time, however, we do not know if there is any long-term persistence of either the vector or vaccine that might account for these observations.

The immunotherapeutic potential of antibodies generated to the O antigen and capsular polysaccharide (CPS) of *B*. *mallei* and *B*. *pseudomallei* has been previously evaluated, and were shown to mediate significant protection in several animal models of glanders and melioidosis. A number of passive immunization studies have been performed using either polyclonal antisera [70,71] or monoclonal antibodies reactive with LPS or capsular polysaccharides of *B*. *mallei* and *B*. *pseudomallei*. In these experiments, mice were passively immunized with either a single or multiple doses of polyclonal or mAb, which were administered intravenously or intraperitoneally. Of note is the observation that in these experiments animals survived or had delayed mortality but sterile immunity was never achieved; bacterial colonization and spleen abscesses was reported [27–30,72,73].

Studies form our group have previously reported the therapeutic potentials of antisera obtained from immunized animals during acute pneumonia [58]. However, in this study we did not observe the anticipated protection in mice passively immunized with sera from vaccine-immunized animals (data not shown). Others have shown that *B*. *thailandensis* is capable of avoiding humoral immunity by invading epithelial cells and growing intracellularly within phagocytes [74]. Thus, active vaccination may be superior therapy for *Burkholderia* infections [28].

We focused our analysis on the antisera induced by intranasal vaccination to examine the efficiency of vaccine immune sera, and to further investigate whether antibodies to O antigen correlates with the protection observed with active immunization. We performed opsonophagocytosis assays using pooled antisera from mice to further confirm the role of anti-LPS antibodies in protection in our model. Efficient and significant killing of *B*. *thailandensis* (>50%) was observed with vaccine serum up to 1:72,900, compared to 1:2,700 with the vector serum (Fig 6). No killing was observed in the absence of vaccine immune serum despite of the presence of complement and phagocytes further confirming the opsonic specificity of the anti-LPS antibodies.

The presence of IgG1, IgG2a, and IgM in the immune serum has been previously correlated with high opsonic activity [75,76]. In our model, we found that anti-*B*. *mallei* O-antigen antibodies of IgG1, IgG2a, and IgG3 subclasses were produced in mice in response to vaccination (Fig 2D). Together with the opsonophagocytosis data, our results strongly suggest that antibodies with high opsonic activity resulted enhanced phagocytosis of extracellular bacteria (Fig 6), which agrees with the previous findings showing that anti-polysaccharide antibodies can enhance opsonophagocytosis of *B*. *pseudomallei* [30,77].

While the intraperitoneal route is commonly used in experimental animal models, the subcutaneous or intranasal route of infection are considered more physiologically relevant model for melioidosis and glanders. In endemic regions and in a potential bio-threat situation, inhalation is an important route of exposure in human melioidosis and glanders infection [1,6]. A strong airway immune response seems to be best achieved through mucosal vaccination; intranasal immunization has proven to be superior in stimulating optimal and protective immune responses to several antigens including LPS at the lung mucosal surface [48,58,78]. Studies of other respiratory infections such as influenza, tuberculosis, and tularemia, have demonstrated that to effectively generate protective mucosal immune responses, the vaccine should be delivered directly to the target mucosa rather than systemically [79–81]. In all studies of melioidosis and glanders, the lung is often associated with severe disease with pneumonia being the most prominent clinical presentation [82–84]. Despite of the fact, very few studies have investigated the role vaccine-mediated protection against respiratory challenge [85].

Vaccination strategies using attenuated *Salmonella* are principally based on the ability the bacteria to persist in antigen-presenting cells during their migration to lymphatic organs of the mucosal immune system where the desired antigen can be processed and presented to naïve T and B cells [86]. It is well established that mucosa-induced antibody secreting cells predominantly home to their induction site [87]. Intranasal immunization is very efficient at inducing humoral and cellular immune responses in the respiratory mucosa and at distant mucosal sites [87,88]. Also, it has been shown that intranasal immunization is more effective in inducing generalized mucosal and systemic immune responses [89]. Therefore, protection against pulmonary melioidosis and glanders should be greatly enhanced following mucosal immunization as opposed to intraperitoneal vaccination [89].

This same vaccine system could be used to deliver other antigens to combat glanders and melioidosis. Immunogenic protein antigens have been recognized by probing a well-characterized *B*. *pseudomallei* protein array platform and comparing sera from healthy control subjects form South East Asia, patients either recovered from or with acute melioidosis [90,91], and a laboratory worker accidentally infected with *B*. *mallei* [92]. A number of proteins were recognized by antibodies from melioidosis and glanders but not control patients. Of these, two proteins (50S ribosomal protein L7/L12 and type 4 pilus biosynthesis protein) were recognized by antibodies from glanders infections as well as melioidosis patients [92]. The ribosomal protein L7/L12 has been previously identified as immunodominant and protective antigen of *Brucella abortus*, the causative of Brucellosis, another major worldwide disease [93]. These data suggest that other potential candidates could also be expressed in *Salmonella* to provide protection against both infections.

In conclusion, our results demonstrate that intranasal delivery of *Salmonella* expressing *B*. *mallei* LPS O antigen elicits a protective immune response at the site of infection. The ability of this vaccine to induce potent humoral- and cell-mediated immunity represents a promising first step towards the development of a platform for potential immunotherapeutics against melioidosis and glanders.
